# Hemolysis in human erythrocytes by *Clostridium perfringens* epsilon toxin requires activation of P2 receptors

**DOI:** 10.1080/21505594.2018.1528842

**Published:** 2018-10-02

**Authors:** Jie Gao, Wenwen Xin, Jing Huang, Bin Ji, Shan Gao, Liang Chen, Lin Kang, Hao Yang, Xin Shen, Baohua Zhao, Jinglin Wang

**Affiliations:** a State Key Laboratory of Pathogen and Biosecurity, Institute of Microbiology and Epidemiology, AMMS, Beijing, China; b College of Life Sciences, Hebei Normal University, Shijiazhuang, China

**Keywords:** *Clostridium perfringens*, ϵ-toxin, erythrocytes, hemolysis, P2 receptors

## Abstract

Epsilon-toxin (ETX) is produced by types B and D strains of *Clostridium perfringens*, which cause fatal enterotoxaemia in sheep, goats and cattle. Previous studies showed that only a restricted number of cell lines are sensitive to ETX and ETX-induced hemolysis has not previously been reported. In this study, the hemolytic ability of ETX was examined using erythrocytes from 10 species including murine, rabbit, sheep, monkey and human. We found that ETX caused hemolysis in human erythrocytes (HC_50_ = 0.2 μM) but not erythrocytes from the other test species. Moreover, the mechanism of ETX-induced hemolysis was further explored. Recent studies showed that some bacterial toxins induce hemolysis through purinergic receptor (P2) activation. Hence, the function of purinergic receptors in ETX-induced hemolysis was tested, and we found that the non-selective P2 receptor antagonists PPADS inhibited ETX-induced lysis of human erythrocytes in a concentration-dependent manner, indicating that ETX-induced hemolysis requires activation of purinergic receptors. P2 receptors comprise seven P2X (P2X1–7) and eight P2Y (P2Y1, P2Y2, P2Y4, P2Y6, and P2Y11–P2Y14) receptor subtypes. The pattern of responsiveness to more selective P2-antagonists implies that both P2Y13 and P2X7 receptors are involved in ETX-induced hemolysis in human species. Furthermore, we demonstrated that extracellular ATP is likely not involved in ETX-induced hemolysis and the activation of P2 receptors. These findings clarified the mechanism of ETX-induced hemolysis and provided new insight into the activities and ETX mode of action.

## Introduction

*Clostridium perfringens* is a Gram-positive, spore-forming, anaerobic, rod-shaped bacterium that carries genes encoding more than 17 exotoxins. It is conventionally categorized into five toxinotypes, A to E, based on carriage of one or more of the major toxin genes (alpha, beta, epsilon, or iota) [1]. *C. perfringens* epsilon toxin (ETX) is mainly produced by *C. perfringens* types B and D [2]. The toxin is the aetiological agent of dysentery in newborn lambs but is also associated with enteritis and enterotoxaemia in goats, calves and foals [3]. ETX is the third most lethal of all clostridial toxins, ranking behind only the *Clostridium botulinum* and *Clostridium tetani* neurotoxins [4]. The fact that the 50% lethal dose (LD_50_) of ETX in mice is 50 ng·kg^−1^ [5] underpins the potential to use this toxin as a bioterrorist weapon; therefore, ETX is regarded as a potential bioterrorism agent by the US Government Centers for Disease Control and Prevention [3].

The relatively inactive secreted prototoxin is converted to the fully active mature toxin by proteases of the host, such as trypsin and chymotrypsin [6], or by the *C. perfringens* λ-protease [5]. Active ETX binds to the intestinal epithelium to induce epithelial permeability in the absence of overt histologic damage [7] and enters the bloodstream. *In vivo*, the toxin appears to target the brain and kidneys, but a relatively restricted number of cell lines are susceptible to the toxin, with most work having been carried out in Madin Darby canine kidney (MDCK) cells [3]. Over the past few decades, a number of cell lines have been tested to identify a suitable *in vitro* model for the study of ETX [3]. The Caucasian renal leiomyoblastoma (G-402) cell line [8], human kidney cell line ACHN [9], and murine renal cortical collecting duct principal cell line mpkCCDcl4 [10] were identified to be toxin-sensitive, albeit to a lesser extent than the MDCK cell line [3].

ETX is considered to be a pore-forming toxin. It was reported that ETX formed a heptameric membrane complex that created ~2-nm-wide pores in the MDCK cell membrane and led to membrane permeabilization, in turn leading to a rapid decrease in intracellular K^+^, an increase in Na^+^ and Cl^−^, and a delayed increase in Ca^2+^ [11]. Many pore-forming toxins could cause hemolysis such as α-hemolysin (HlyA) from *Escherichia coli* [12] and α-toxin from *Staphylococcus aureus* [13]. However, ETX-induced hemolysis has not previously been reported. We hypothesized that ETX may cause hemolysis in red blood cells (RBCs). Hence, the hemolytic ability of ETX was examined using erythrocytes from many species, such as murine, rabbit, sheep, equine, monkey and humans. Surprisingly, we found that ETX causes hemolysis in human erythrocytes but not erythrocytes from the other test species. This finding led us to investigate the mechanism of ETX-induced hemolysis.

Previous studies showed that purinergic (P2) receptor activation is involved in some bacterial toxin-induced hemolysis of erythrocytes. For example, hemolysis induced by HlyA from *E. coli* [12] and α-toxin from *S. aureus* [13] requires P2X receptor activation. We hypothesized that ETX-induced hemolysis may require activation of P2 receptors [12–14]. Purinergic receptors that respond to extracellular nucleotides are termed P2 receptors, and comprise P2X and P2Y receptor subtypes [15,16]. P2 receptors are activated by extracellular adenosine triphosphate (ATP) and other nucleotides [16]. In mammals, seven P2X receptor subtypes exist (P2X1–P2X7) [17]. P2X receptors are trimeric ATP-gated cation channels that mediate the rapid flux of Na^+^, K^+^, Ca^2+^, and organic ions [16,17]. P2Y receptors modulate several signaling events including adenylyl cyclase, phospholipase C, and ion channel activation [16,18]. P2 receptors are present on all blood cells [19]. It is reported that some pore-forming toxins require purinergic signaling to elicit their toxicity [16].

In the present study, we investigated the characteristics of ETX-induced hemolysis. Also, the function of purinergic receptors in ETX-induced hemolysis of human erythrocytes was tested using various antagonists. Furthermore, the role of ATP in the activation of P2 receptors was determined and consequently the mechanism of ETX-induced hemolysis was clarified.

## Results

### ETX causes hemolysis in human erythrocytes

**Figure 1.**
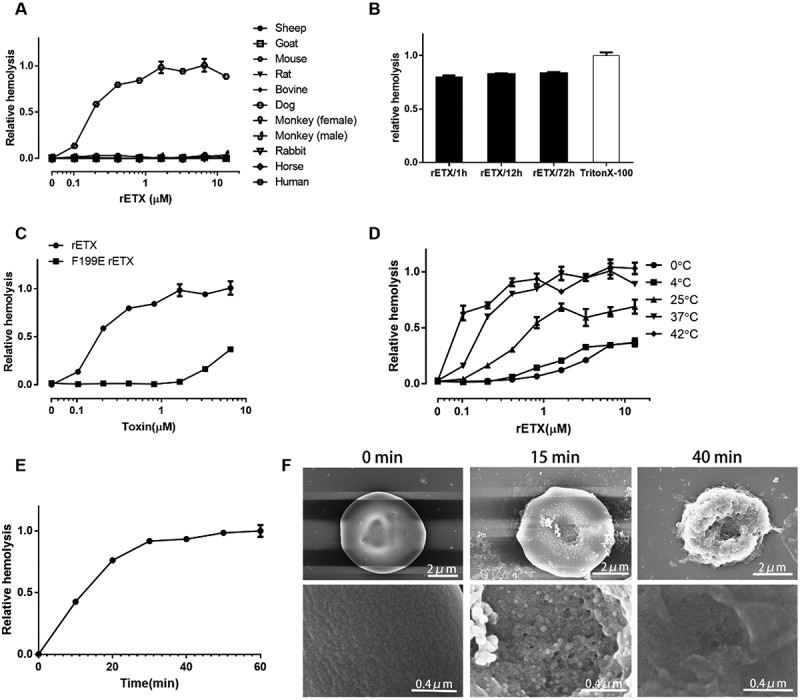
ETX induces hemolysis in human erythrocytes. Human erythrocytes in 3.3% solution were used. (a) Hemolytic ability of rETX was tested using erythrocytes from various animal sources and humans. The various erythrocytes were incubated with rETX (0–13 μM) for 60 min at 37°C. Hemolysis value corresponding to 6.5 μM ETX is defined as 1. (b) The maximal extent of hemolysis induced by ETX. Human erythrocytes in 3.3% solution were incubated with rETX (30 μM) for 1 h, 12 h, or 72 h at 37°C and 1% TritonX-100 for 1 h at 37°C. Hemolysis of erythrocytes treated with Triton X-100 is defined as 1. (c) Extent of hemolysis in human erythrocytes induced by the mutant rETX^F199E^. Human erythrocytes were incubated with serial dilutions of purified rETX and rETX^F199E^. Hemolysis value corresponding to 6.5 μM ETX is defined as 1. (d) Hemolytic ability of rETX was tested at various temperatures. Human erythrocytes were incubated with rETX (0–13 μM) for 60 min at 0°C, 4°C, 25°C, 37°C, and 42°C. Hemolysis value corresponding to 6.5 μM ETX is defined as 1. (e) Extent of hemolysis with time. The erythrocytes were incubated with rETX (0.2 μM) from 0 to 60 min at 37°C. Values are the mean ± SD (n = 3). Hemolysis value corresponding to 60 min is defined as 1. (f) Scanning electron microscopy images of human erythrocytes treated with rETX. Human erythrocytes were incubated with rETX (0.2 μM) for 0, 15, or 40 min at 37°C and then attached to a coverslip.

Recombinant ETX (rETX) with 6× His-tag was expressed in the *E. coli* BL21 (DE3) strain and purified using a Ni^2+^ chelating affinity chromatography resin column. A high-purity of rETX protein was obtained. No unwanted band was detected in the gel (S1 Fig). The hemolytic ability of rETX was examined using erythrocytes from various sources including murine, rabbit, sheep, goat, cattle, equine, dog, monkey, and human. The erythrocyte suspension (final concentration of 3.3%) from various sources was incubated with purified rETX (final concentration of 0.03–30 μM). The result showed that rETX lysed human erythrocytes, but not erythrocytes from the other test species (Figure 1(a)). The effect of hemolysis increased with the concentration of rETX (Figure 1(a)). The maximal concentration (30 μM) of rETX lysed ~ 80% human erythrocytes after 60 min incubation at 37°C (Figure 1(b)). Complete hemolysis was not observed, with the maximal level of hemolysis remaining around 80% even after 12–72 h of incubation with a high concentration of ETX (~30 μM) at 37°C (Figure 1(b)).

To verify that the observed hemolysis was induced by rETX and not contaminating proteins from the engineered *E. coli* strain, we tested the effect of the attenuated rETX mutant rETX^F199E^ (the 199th amino acid residue of phenylalanine was replaced by a glutamic acid) [20] on the hemolysis of human erythrocytes. rETX^F199E^ was expressed and purified using the same method as rETX. rETX^F199E^ exhibited an attenuated hemolytic ability (Figure 1(c)), indicating that the hemolysis could in fact be ascribed to rETX. rETX was used in the following experiments.

Temperature plays an important role in ETX-induced hemolysis (Figure 1(d)). Hemolysis was mostly inhibited at 4°C or a lower temperature. The level of hemolysis at 37°C resembled that at 42°C. The concentration of rETX required for 50% hemolysis (HC_50_) after 60 min incubation at 37°C was 0.2 μM. In all subsequent experiments, 0.2 μM of rETX was therefore used. ETX-induced hemolysis is also time-dependent, with the level of hemolysis increasing with time. It took approximately 30 min for human erythrocytes to reach stable phase (Figure 1(e)).

To further survey the morphological effects of toxins on human erythrocytes, scanning electron microscopy was performed (Figure 1(f)). RBCs in the control group showed a biconcave shape and a smooth surface. By contrast, sags and crests were observed on the surface of ETX-treated human erythrocytes, which gradually increased with time.

### Etx-induced hemolysis requires activation of purinergic receptors

**Figure 2.**
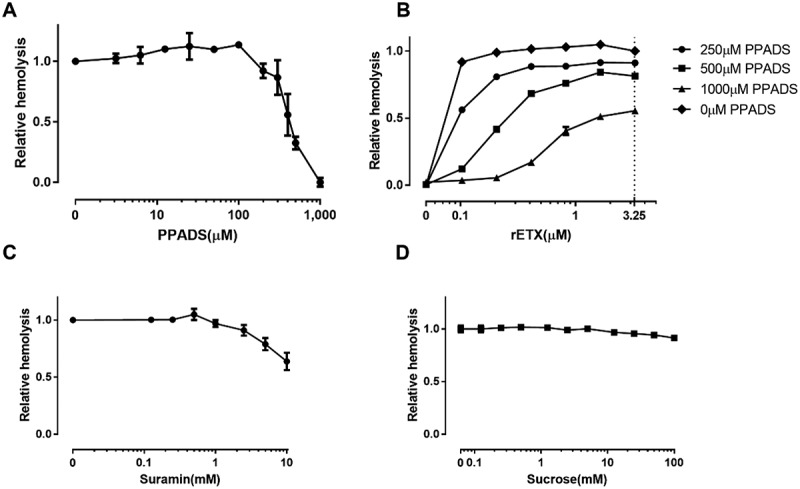
ETX-induced hemolysis of human erythrocytes is inhibited by non-selective purinergic-receptor (P2) antagonists. (a) Effect of the non-selective P2 receptor antagonist PPADS on ETX-induced lysis of erythrocytes. Hemolysis value corresponding to 0 μM PPADS is defined as 1. (b) Concentration-response relationship of PPADS at increasing concentration of purified rETX (0–3.25) in human erythrocytes. Hemolysis value corresponding to 1.625 μM ETX is defined as 1. (c) The effect of suramin on ETX-induced hemolysis of human erythrocytes. Hemolysis value corresponding to 0 mM suramin is defined as 1. (d) The effect of increasing concentrations of sucrose on the hemolysis of human erythrocytes. Values are the mean ± SD (n = 3). Hemolysis value corresponding to 0 mM sucrose is defined as 1.

It has been reported that P2 receptor activation is involved in toxin-induced hemolysis of erythrocytes, such as the hemolysis induced by *E. coli* HlyA and *S. aureus* α-toxin [12,13]. We hypothesized that ETX-induced hemolysis requires activation of P2 receptors and investigated whether P2 receptor antagonists affected ETX-induced hemolysis. We found that the non-selective P2 receptor antagonist PPADS decreased hemolysis induced by ETX in human erythrocytes in a concentration-dependent manner (Figure 2(a)). The EC_50_ (concentration for 50% of the maximal effect) value for PPADS was 519 μM. This finding was substantiated for a wide range of ETX concentrations (Figure 2(b)). The concentration response relationship was compatible with competitive antagonism, and it should be noted that the effect of even 3.3 μM of toxin was reduced to approximately 50% by the P2 receptor blocker (1000 μM PPADS). Thus, P2 receptor activation seems to be involved in ETX-induced hemolysis. The non-selective P2 receptor antagonist suramin also decreased ETX-induced hemolysis in a concentration-dependent manner (Figure 2(c)).

To evaluate whether the effect of the purinergic antagonist on hemolysis was merely a result of increased osmolality, we tested the effect of extracellular sucrose on ETX-induced hemolysis. Surprisingly, sucrose did not decrease hemolysis, even at a concentration of 100 mM (Figure 2(d)). This suggested that ETX-induced hemolysis is not related to osmolality. Considering that the concentrations of the antagonists used in this study never exceeded 10 mM, the effect cannot be the result of increased osmolality.

### P2Y13 and P2X7 are involved in ETX-induced hemolysis

Erythrocytes express various types of P2 receptors. The P2 receptors that have been confirmed to be expressed in mature human erythrocytes include P2Y1, P2Y2, P2Y13, P2X1, P2X2, and P2X7 [16]. We tested whether these receptors were responsible for ETX-induced hemolysis using various antagonists of the receptors (Table 1). As there are no specific antagonists for P2Y2 receptor and P2X2 receptor and low predicted expression levels of P2Y2 receptor on human erythrocytes [16,21], the effect of P2Y2 and P2X2 receptors were not examined.

**Table 1.** Antagonists tested.

Antagonists	Target	Effect on ETX-induced hemolysis
PPADS	P2 receptors	√
Suramin	P2 receptors	√
Evans blue	P2X	√
MRS2179	P2Y1	×
MRS2211	P2Y13	√
NF449	P2X1	×
NF023	P2X1	×
BBG	P2X7/P2X1	√
MRS2159	P2X7/P2X1	√
Mg^2+^	P2X7	√
OxATP	P2X7/P2X1	√
KN-62	P2X7/P2X1	√
A438079	P2X7	√
Carbenoxolone	Pannexin 1	×
Mefloquine	Pannexin 1	×

**Figure 3.**
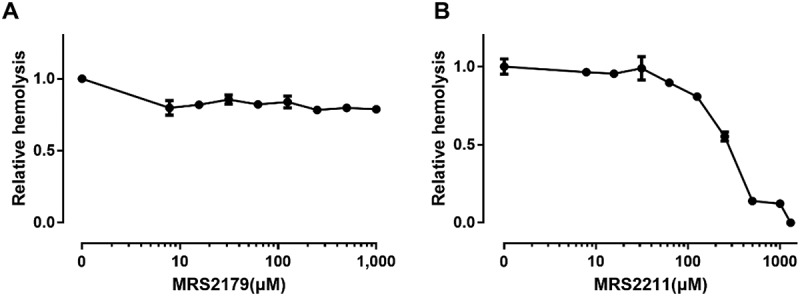
Effect of P2Y antagonists on ETX-induced hemolysis. (a) Erythrocytes were incubated with rETX (0.2 μM) and increasing concentrations of MRS2179 (P2Y1 receptor antagonist). Hemolysis value corresponding to 0 μM MRS2179 is defined as 1. (b) Erythrocytes were incubated with rETX (0.2 μM) and increasing concentrations of MRS2211 (P2Y13 receptor antagonist). Values are the mean ± SD (n = 3). Hemolysis value corresponding to 0 μM MRS2211 is defined as 1.

Functional P2Y1 plays a role in promoting malaria parasite development in human erythrocytes [22]. Our results showed that the P2Y1 receptor antagonist MRS2179 was inefficient in the inhibition of ETX-induced hemolysis (Figure 3(a)) at concentrations up to 1000 μM. To investigate the role of P2Y13 receptor in ETX-induced hemolysis we tested the antagonist MRS2211, which has been reported to display some selectivity toward the P2Y13 receptor [12,23]. MRS2211 decreased hemolysis caused by ETX in a concentration-dependent manner. MRS2211 decreased ETX-induced hemolysis to ~50% at a concentration of 243 μM (Figure 3(b)). Taken together, these results indicated that the P2Y13 receptor is involved in ETX-induced hemolysis.

**Figure 4.**
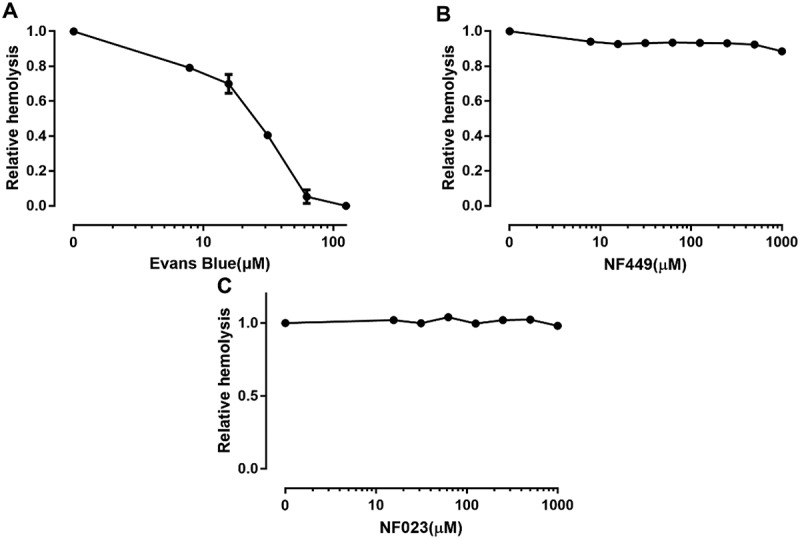
Effect of P2X antagonists on ETX-induced hemolysis. (a) Erythrocytes were incubated with rETX and increasing concentrations of Evans Blue (non-selective P2X receptor antagonist). Hemolysis value corresponding to 0 μM Evans Blue is defined as 1. (b, c) Erythrocytes were incubated with rETX and increasing concentrations of NF449 and NF023 (P2X1 receptor antagonists). Values are the mean ± SD (n = 3). Hemolysis value corresponding to 0 μM NF449 or NF023 is defined as 1.

Having tested P2Y receptor antagonists we then moved onto testing non-selective and selective P2X receptor antagonists. Figure 4(a) shows that the non-selective blocker of P2X receptors, Evans blue, potently reduced ETX-induced hemolysis (EC_50_: ~1.2 μM), suggesting that a P2X receptor is involved in ETX-induced hemolysis. Of the P2X-receptors expressed in erythrocytes, we regarded P2X7 and P2X1 as the most likely mediators of ETX-induced hemolysis according to previous studies [12,16]. P2X1 and P2X7 mediate bacterial toxin-induced lysis of RBCs from various species [12–14].

We tested the P2X1 selective antagonists NF449 and NF023 [24]. NF449, a suramin derivative, is 100,000-fold more potent in blocking P2X1 than P2X7 receptors [25]. NF449 and NF023 did not affect ETX-induced hemolysis (Figure 4(b,c)). These results indicated that P2X1 receptor is unlikely relevant to ETX-induced hemolysis.

**Figure 5.**
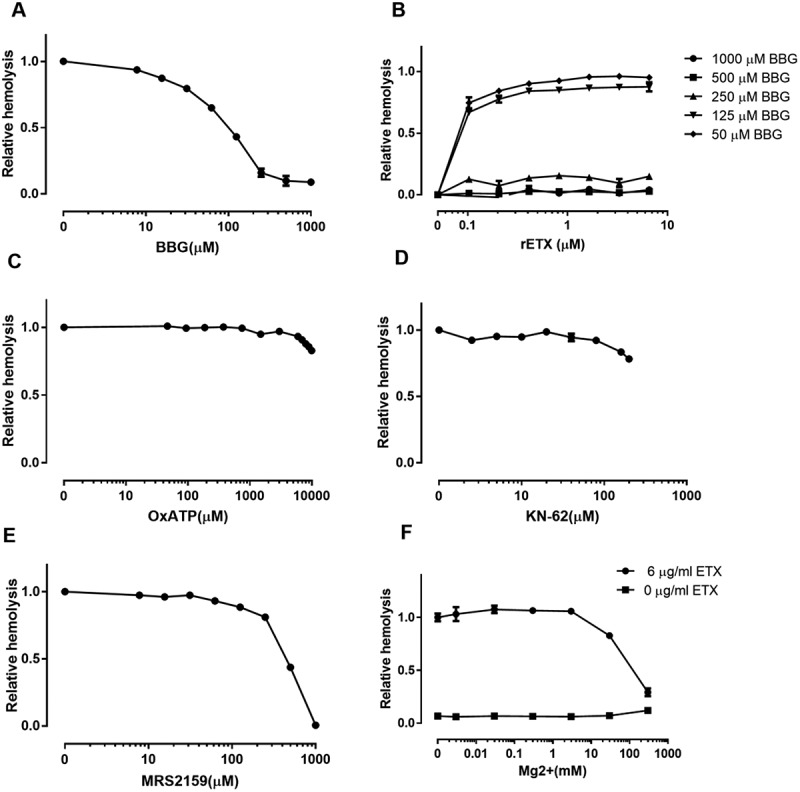
ETX-induced hemolysis is inhibited by certain P2X7 receptor antagonists. Hemolysis induced by ETX was potently reduced by increasing concentrations of (a) BBG, (e) MRS2159, and (f) Mg^2+^. (b) Concentration-dependent effect of BBG at various concentrations of purified rETX. ETX-induced hemolysis was slightly reduced by high concentrations of (c) OxATP, and (d) KN-62. Values are the mean ± SD (n = 3). Hemolysis value corresponding to 0 μM each antagonist is defined as 1.

To test whether P2X7 receptors participate in ETX-induced hemolysis, we used antagonists with relative selectivity for P2X7: Brilliant Blue G (BBG), ATP-2′, 3′-dialdehyde (OxATP), MRS2159 [26], KN-62 [12], Mg^2+^ [25], and A438079 [12]. BBG decreased hemolysis in a concentration-dependent manner with an EC_50_ value of 90 μM (Figure 5(a)). The protection against hemolysis by BBG was again substantiated across the whole concentration range of ETX in human erythrocytes using BBG as an example of a P2X7 antagonist (Figure 5(b)). Moreover, the antagonist (above 500 μM) had a substantial effect (complete inhibition) on ETX-induced hemolysis even at ETX concentrations (6.7 μM) that produced maximal hemolysis.

By contrast to BBG, other P2X7 receptor antagonists OxATP and KN-62 slightly decreased ETX-induced hemolysis by 20% at the maximal concentrations tested (Figure 5(c,d)). The novel, selective, competitive P2X7 receptor antagonist A438079 slightly reduced hemolysis in human erythrocytes by 5% (S2 Fig).

MRS2159, which is generally considered a P2X1 antagonist, is also a potent antagonist of P2X7 with an IC_50_ of at least 1 log lower than that of A438079 [26]. MRS2159 potently inhibited hemolysis in human erythrocytes in a concentration-dependent manner (EC_50_: ~349 μM) (Figure 5(e)).

As P2X1 and P2X7 share similar inhibitor profiles for BBG, KN-62, OxATP and MRS2159 [12,25,26], we cannot exclude the possibility that P2X1 receptor is partially involved in ETX-induced hemolysis in human erythrocytes. A previous study showed that Mg^2+^ ions were effective in suppressing the P2X7 current component in P2X1/7 co-expressing oocytes, whereas the P2X1 current component remained relatively unaltered [25]. Therefore, Mg^2+^ ions were used to be a high selective antagonist of P2X7. Our results showed that Mg^2+^ ions inhibited the ETX-induced hemolysis in a concentration-dependent manner (EC_50_: ~252 mM) (Figure 5(f)), indicating that the P2X7 receptor rather than the P2X1 receptor is involved in ETX-induced hemolysis.

### ETX-induced hemolysis is not mediated by ATP

**Figure 6.**
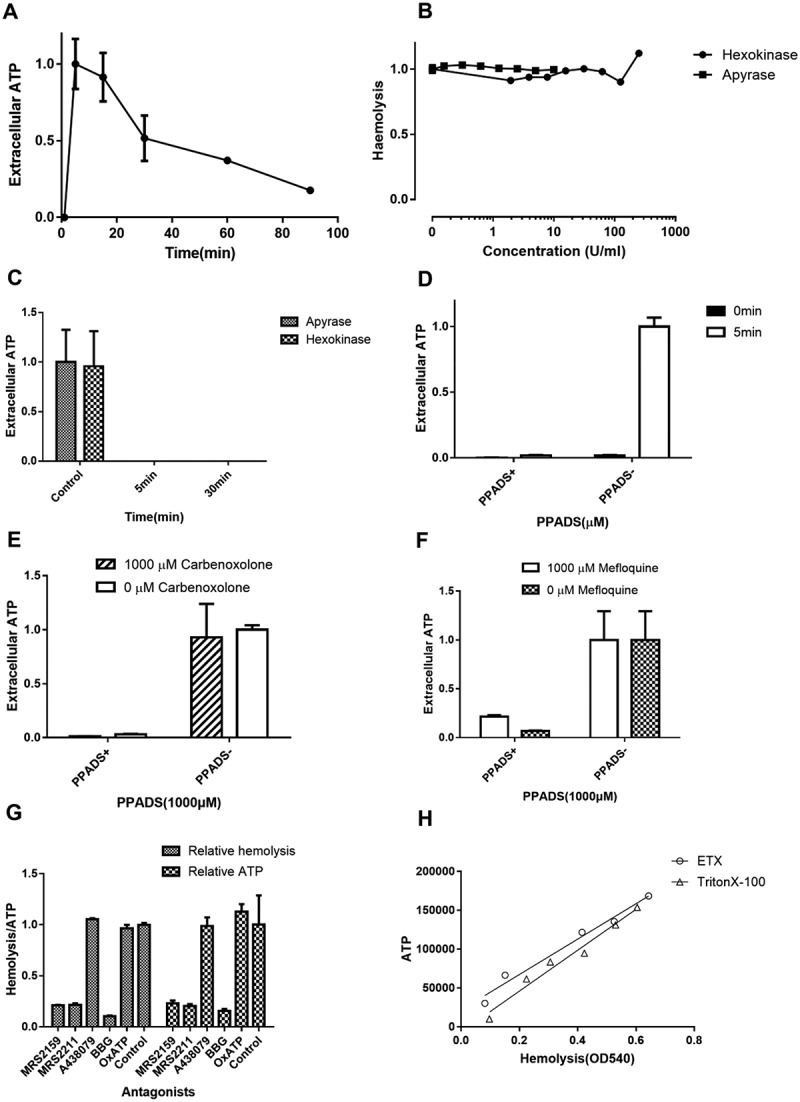
Relationship between ETX-induced hemolysis of human erythrocytes and extracellular ATP. (a) Adding rETX (0.2 μM) to human erythrocytes caused a rapid increase in extracellular ATP content from 0–5 min, and then a time-dependent decrease. ATP value corresponding to 5 min is defined as 1. (b) The relative hemolysis of human erythrocytes incubated with increasing concentrations of the ATP scavenging enzymes apyrase and hexokinase and rETX (0.2 μM). Hemolysis value corresponding to 0 μM hexokinase or apyrase is defined as 1. (c) Extracellular ATP content of human erythrocytes after incubation with apyrase (1.25 U/ml) and hexokinase (31.25 U/ml) and rETX (0.2 μM) for 5 and 30 min. (d) The extracellular ATP of the human erythrocytes treated with rETX (0.2 μM) for 0 and 5 min with or without 1000 μM PPADS. (e, f) The effect of pannexin 1 antagonists (e) carbenoxolone (p > 0.05, Student’s t-test) and (f) mefloquine (p > 0.05, Student’s t-test) on the extracellular ATP content of human erythrocytes exposed to rETX (0.2 μM) with or without 1000 μM PPADS. (g) Extent of hemolysis and ATP release induced by ETX with antagonists. Pair-wise measurements of hemolysis and ATP release after incubation for 5 min. (h) Correlation between the extent of hemolysis and ATP release induced by ETX. Triton X-100 induced hemolysis and ATP release were compared with those induced by ETX. Various concentrations of ETX (0 ~ 0.94 μM) and TritonX-100 (0 ~ 0.00885%) were used. Paired values were fitted by linear regression using the GraphPad Prism 7 software. Correlation coefficient R = 0.978 (ETX) and 0.972 (Triton X-100). Values are the mean ± SD (n = 3).

Recent findings indicated that *E. coli* HlyA and *Aggregatibacter actinomycetemcomitans* leukotoxin A induce ATP release from RBCs by forming toxin pores, which then acts on P2X1 and P2X7 to mediate hemolysis [27]. Therefore, we tested the role of ATP in ETX-induced hemolysis. A large amount of ATP was released at the initial stage of hemolysis, peaking after 5 min (Figure 6(a)). The ATP scavenging enzymes apyrase and hexokinase continuously degrade extracellular ATP, and consequently might impair the activation of P2 receptors. However, we found that these enzymes had little effect on ETX-induced hemolysis (Figure 6(b)), even when extracellular ATP was totally abolished by the enzymes (Figure 6(c)), suggesting that P2 receptors are activated by something other than ATP.

Additionally, we found that PPADS (1.0 mM) could abolish ATP release by ETX-treated RBCs (Figure 6(d)), suggesting that the release of ATP is caused by ETX-induced hemolysis. This raises the question of how ATP is released. Pannexin1 is expressed in human RBCs and has recently been suggested to operate as the ATP release channel in erythrocytes [12,28,29]. To assess the effect of pannexin 1 on ATP release, carbenoxolone and mefloquine were used as antagonists for pannexin 1 [30,31]. The results showed that inhibition of pannexin 1 did not decrease the release of ATP (Figure 6(e,f)), indicating that ATP was not released via pannexin1.

We hypothesized that ATP release is likely the result of hemolysis and that ATP is released when erythrocytes are disrupted by activation of P2 receptors. Many antagonists such as MRS2159, MRS2211, A438079, BBG, and OxATP, were employed to investigate the relationship between ATP release and hemolysis of erythrocytes (Figure 6(g)). Furthermore, Triton X-100-induced hemolysis and ATP release were compared with those induced by ETX. Each sample was investigated by paired measurement of free hemoglobin and ATP. We found that the extent of ATP release tightly correlated with the extent of hemolysis (Figure 6(g,h)). These results indicated that hemolysis is responsible for most, if not all, ATP release.

### Pannexin 1 is not involved in ETX-induced hemolysis

**Figure 7.**
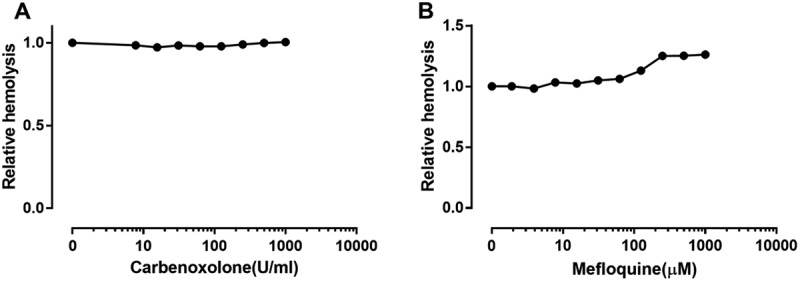
Effect of pannexin 1 antagonists on ETX-induced hemolysis in human erythrocytes. The erythrocytes in 3.3% solution were incubated with rETX (0.2 μM) and (a) carbenoxolone or (b) mefloquine for 60 min at 37°C. Values are the mean ± SD (n = 3). Hemolysis value corresponding to 0 μM of each antagonist is defined as 1.

The P2X7 receptor has been reported to interact with the channel protein pannexin 1 [32], and this complex creates a sizeable pore permeable to larger molecules such as ethidium bromide [33]. To assess the effect of pannexin1 on ETX-induced hemolysis, carbenoxolone and mefloquine were used as antagonists for pannexin 1. The results showed that carbenoxolone (Figure 7(a)) had no effect on ETX-induced hemolysis and mefloquine (Figure 7(b)) slightly increased ETX-induced hemolysis by 25%, indicating that pannexin 1 is less involved in ETX-induced hemolysis.

## Discussion

ETX was first described by Wilsdon over 70 years ago [34]. ETX has been known to be a pore-forming toxin since 2001 [11]. Over the past few decades, the biological activity of ETX was tested and examined using a number of cell lines originating from various organs including the kidney, bone, brain, skin, and the respiratory and intestinal tracts [8]. However, ETX-induced hemolysis has not been investigated to any extent. During our initial tests of the biological activity of ETX, we discovered that ETX causes hemolysis exclusively in human erythrocytes. This finding was surprising and interesting.

The characteristics of ETX-induced hemolysis were determined. The morphological effects of ETX-induced hemolysis in RBCs appeared to resemble those in MDCK cells. Similar sags and crests were observed on the surface of RBCs as those on ETX-treated MDCK cells [20]. A stable complex was detected in the ETX-treated membrane of both RBCs and MDCK cells (S3 Fig). However, the complex formed on human RBCs was larger than that on MDCK cells (S3 Fig), indicating that a membrane protein may also be involved in the formation of the complex in human RBCs. ETX-induced hemolysis is an incomplete hemolysis. Similar phenomenon is also observed in MDCK cells. ETX was able to cause 90% death of MDCK cells (Survival percent of 10%) at maximal concentration (~33 μM, S4 Fig). Temperature plays an important role in ETX-induced hemolysis, as also shown in MDCK cells [35]. This suggested that ETX-induced hemolysis is probably associated with the fluidity of the membrane [36].

The preferred hemolysis in human RBCs indicated that ETX likely causes hemolysis in a receptor-dependent manner. This was substantiated by an ETX binding assay. The binding assay showed that rETX could bind to the surface of human erythrocytes compared with sheep erythrocytes (S5 Fig). Taken together, these data suggested that there was a receptor exclusively expressed on the surface of human RBCs. ETX is a pore-forming toxin; therefore, changes in plasma osmolality commonly induced by toxin-formed pores are likely to be the main reason for ETX-induced hemolysis. However, we found that a high concentration of sucrose did not affect hemolysis, indicating that osmolality plays little role in ETX-induced hemolysis. This raises a new question: what is the mechanism of ETX-induced hemolysis? Therefore, we further investigated whether P2 receptors are involved in ETX-induced hemolysis.

We found that ETX-induced hemolysis is blocked by various inhibitors of purinergic signaling. PPADS, a non-selective purinergic antagonist, entirely eliminates ETX-induced hemolysis. Suramin, another non-selective P2 antagonist, reduces ETX-induced hemolysis with less efficiency. Taken together, these data imply a central role for P2 receptors in ETX-induced hemolysis. This finding led us to test a series of antagonists to find out which P2 receptor(s) are involved in ETX-induced hemolysis. The results showed that P2Y13 and P2X7 are mainly involved in ETX-induced hemolysis.

Functional P2Y13 is present on RBCs, where it negatively regulates ATP release from these cells [21]. Activation of this receptor by ADP impairs the release of ATP from human RBCs. P2Y13 is not reported to be relevant to hemolysis. A previous study also found that an antagonist of P2Y13 potently inhibited HlyA-induced hemolysis [12]. However, Skals and coworkers suggested that P2Y13 was not likely to be involved in HlyA-induced hemolysis since this contradicts the results with hexokinase [12], which inhibited HlyA-induced hemolysis. Hexokinase could degrade ATP to ADP, which could stimulate the ADP-sensitive P2Y13 receptor. No such contradiction was observed with our results (Figure 6(b)), therefore, we concluded that P2Y13 is involved in ETX-induced hemolysis. However, we cannot exclude the possibility that the inhibition exerted by MRS2211 is mediated through another P2 receptor.

The P2X1 and P2X7 receptors are known to undergo transition to a greater permeability state, which eventually leads to lysis in certain cells [32]. P2X1 and P2X7 were also reported to be involved in HlyA-induced hemolysis [12]. Purinergic P2X1 and P2X7 receptors are co-expressed in human RBCs. There are a restricted number of selective antagonists for the differentiation of P2X1 and P2X7 subtypes. NF449 and NF023 are demonstrated to be highly selective antagonists of P2X1 but not P2X7. It was reported that Mg^2+^ ions were effective in suppressing the P2X7 current component but left the P2X1 current component relatively unaltered [25]. Taken together, these results of this study indicated that P2X7 rather than P2X1 is involved in ETX-induced hemolysis. It is not clear why only some of the antagonists of P2X7 are effective. It may be because the affinity of the antagonists varies and there is competition between antagonists and ETX.

**Figure 8.**
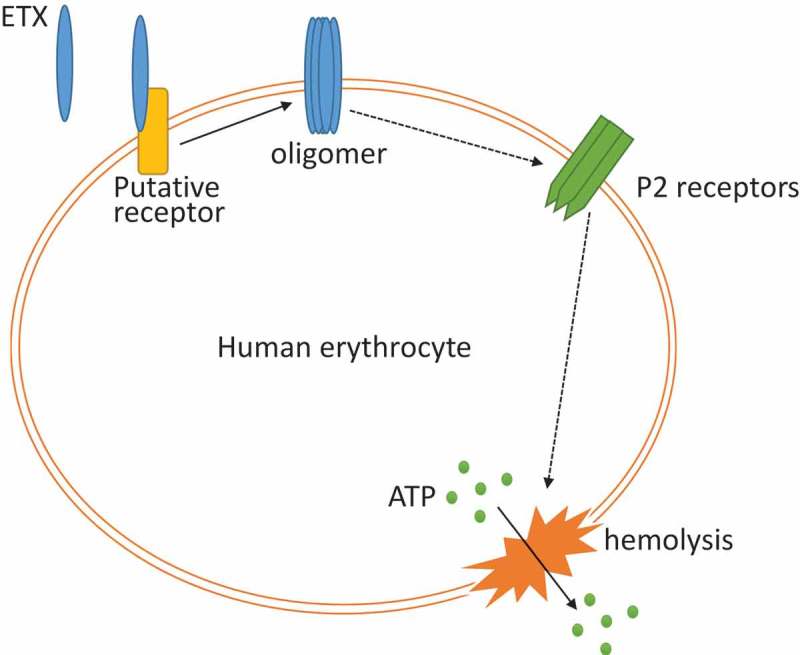
Inferred model for ETX-induced hemolysis. ETX first bound to membrane of human erythrocytes and formed pores; secondly, a series of signal transduction occurred and led to P2 receptor’s activation; thirdly, activation of P2 receptors led to hemolysis, which caused the release of ATP.

Studies reported that HlyA from *E. coli* and leukotoxin A from *A. actinomycetemcomitans* induce ATP release from RBCs by forming toxin pores. Released ATP then acts on P2X1 and P2X7 to mediate hemolysis [12,14,16]. ATP as a mediator, plays an important role in HlyA- and leukotoxin A-induced hemolysis. ATP was found to be released from ETX-treated RBCs. However, our results indicated that the ATP-scavengers apyrase and hexokinase could not inhibit ETX-induced hemolysis. It therefore appeared that ATP was not involved in the activation of P2 receptors. This may also explain why OxATP was not effective in inhibiting ETX-induced hemolysis. Since ATP and ATP derivatives share structural similarities, ATP derivatives such as OxATP may potentially impair P2X7 activation by binding to the ATP binding site. OxATP cannot impair P2X7 activation if ATP is not involved in P2X7 activation. We also demonstrated that the release of ATP was caused by hemolysis rather than ETX-formed pores or pannexin 1. In addition, we found that the extent of ATP release tightly correlated with the extent of hemolysis (Figure 6(h)). Taken together, we concluded that ATP release is the result of ETX-induced hemolysis rather than a mediator of ETX-induced hemolysis (Figure 8). Hence, the mechanism of ETX-induced hemolysis is different from those of HlyA- and leukotoxin A-induced hemolysis. It was also reported that hemolysis is a primary ATP-release mechanism in human erythrocytes stimulated by several stimuli, including shear stress and hypoxia [37].

The mechanism by which P2 receptor activation plays a role in ETX-induced hemolysis remains unclear. One possibility is that ETX binds to and activate P2 receptors. Another possibility is that ETX interacts with P2 receptors after binding to cells, which is mediated by another receptor. This could explain why ETX exclusively lyses human RBCs, since P2 receptors are widely expressed in the RBCs of many species that are resistant to ETX-induced hemolysis. An inferred mode of ETX-induced hemolysis is shown in Figure 8. Further studies are required to investigate the role of P2 receptors in ETX-induced hemolysis.

Previous studies suggested that in MDCK cells exposed to ETX, a large membrane complex formed on the cell surface [38], leading to pore formation, efflux of K^+^, influx of Na^+^, Ca^2+^, and Cl^−^ ions, and finally rapid cell death [3,11]. In ETX-treated RBCs, we observed an increase of intracellular Ca^2+^, which could be mostly inhibited by an antagonist of P2 receptors (S6 Fig). This indicated that activation of P2 receptors was responsible for the majority of the influx of Ca^2+^. P2X7 receptors are most highly expressed in immune and epithelial cells [39]. We therefore inferred that the activation of P2 receptors may be involved in ETX-induced death of MDCK cells. The efflux of K^+^, and the influx of Na^+^ and Ca^2+^, are likely the result of activation of P2X receptors since P2X receptors are non-selective cation channels [40]. However, antagonists of P2 receptors did not have any effect on the inhibition of ETX-induced death in MDCK cells (S7 Fig). Therefore, P2 receptors are less likely to be involved in ETX-induced death in MDCK cells. However, it has previously been reported that one or several large blebs surround ETX-treated MDCK cells [38]. This phenotype resembles that of HEK-293 cells with P2X7 activation [39]. In fact, the activation of P2X7 can lead to different phenotypes, such as membrane blebbing and cell death by apoptosis or lysis/necrosis, depending on the cell type [41]. Taken together, there is still a possibility that P2 receptors are involved in ETX-induced death in MDCK cells and other cell types.

Our results suggested that pannexin 1 does not play a significant role in P2X7-mediated hemolysis. A previous study showed that pannexin 1 is part of the pore-forming unit of the P2X7 receptor death complex [32]. Furthermore, pannexin 1 is involved in HlyA-induced hemolysis, but the P2X7-pannexin 1 complex is not an absolute requirement for the hemolysis [12]. Taken together, it is clear that the P2X7-pannexin 1 complex is not involved in ETX-induced hemolysis.

Little is known about the effect of ETX on human health. There are only two case reports of illness in humans related to the epsilon toxin and these were reported in 1955 [42]. Considering that some human cell lines (G402 and ACHN) are sensitive to ETX, ETX is potentially toxic to humans [42]. ETX-induced hemolysis in human erythrocytes provides further evidence of the potential toxicity of ETX in humans. Furthermore, ETX probably causes more severe disease in humans than animals since ETX cannot induce hemolysis in animals. In addition, given the potent effect of antagonists in the inhibition of ETX-induced hemolysis, antagonists of P2 receptors are potential drug candidates to treat ETX-induced disease.

In conclusion, we show that ETX induces hemolysis in human erythrocytes but not in erythrocytes from the 10 animals tested in our study. We also confirmed that ETX-induced hemolysis in human erythrocytes requires the activation of P2 receptors, mainly including P2Y13 and P2X7. Moreover, we demonstrated that ATP is likely not involved in the activation of P2 receptors, and that ATP is the result of ETX-induced hemolysis rather than a mediator of ETX-induced hemolysis. These findings clarified the mechanism of ETX-induced hemolysis and provided new insights into the mode of action of ETX. The mechanism of ETX-induced hemolysis is different from hemolysis reported previously for other pore-forming toxins. However, the mechanism by which ETX activates P2 receptors and the mechanism of ETX-induced hemolysis remains unclear.

## Materials and methods

### Materials

P2 receptor antagonists: Pyridoxal phosphate-6-azo (benzene-2,4-disulfonic acid) tetrasodium salt hydrate (PPADS), MRS2211, MRS2179, MRS2159, A438079, Brilliant Blue G, ATP-2′, 3′-dialdehyde (OxATP), niflumix acid, carbenoxolone disodium salt, and apyrase were purchased from Sigma-Aldrich (St. Louis, MO, United States). NF449, NF023, and Evans Blue tetrasodium salt were purchased from Tocris Bioscience (Abingdon, United Kingdom). Suramin hexasodium salt was purchased from Aladdin (Shanghai, China). Anti-His monoclonal antibody was purchased from EARTHOX Life Sciences (Millbrae, CA, United States). Anti-ETX monoclonal antibody was developed previously by colleagues in our laboratory. HRP-coupled goat anti-mouse IgG antibody was purchased from TransGen Biotech (Beijing, China). The ATP assay kit was purchased from Calbiochem-Merck (Darmstadt, Germany). MTS (3-(4, 5-dimethylthiazol-2-yl) -5(3-carboxymethoxyphenyl) -2-(4-sulfopheny)-2H-tetrazolium, inner salt) was purchased from Promega Corporation (Madison, WI, United States).

### Preparation of erythrocytes

Human blood was collected by venopuncture from healthy volunteers using evacuated blood collection tubes containing EDTA-2K. The blood of mice, rats, sheep, bovine, rabbits, monkeys, horses, dogs and goats were collected, then the erythrocytes were washed three times in 0.01 M PBS (1,000 g, 4°C, 5 min). The “buffy coat” was removed and isolated erythrocytes were packed by centrifugation at 1,000 g for 10 min at 4°C.

### Preparation of recombinant toxins

Our laboratory constructed the recombinant plasmid pTIG-his-etx, which expressed rETX (without 22 C-terminal and 13 N-terminal sequences) and were transferred into *E. coli* BL21 (DE3) cells. The bacteria were grown in 5.0 ml of sterile lysogeny broth (LB) medium and incubated for 6 h with constant swirling (37°C, 180 rpm). Then the culture was transferred to 500 ml of sterile LB medium with Amp^+^ (100 μg/ml) and incubated for 4.5 h at 37°C with constant swirling (180 rpm) to exponential growth phase (measured as the OD_600_). IPTG at a final concentration of 0.5 mM was used to induce the expression of rETX. Then, the medium was cultured in a shaker (16°C, 180 rpm) overnight. The following morning, cultures were centrifuged (8000 rpm, 4°C, 15 min) to pellet the bacteria.

To purify the ETX, which contained a C-terminal His-Tag, the pellet was re-suspended in lysis buffer and the bacteria were lysed by sonication on ice. The lysate was centrifuged at 11,000 rpm for 15 min at 4°C. The clarified supernatant was purified using an Ni^2+^ affinity chromatography column (GE Healthcare, Pittsburgh, PA, United States) as previously described [43,44]. The purified proteins were analyzed by 12% SDS-PAGE.

### Measurements of the hemolytic activity

Washed RBCs were resuspended in 0.01 M PBS to produce a 5% erythrocyte solution. The hemolytic activity was measured spectrophotometrically at 540 nm. In the hemolytic assay, purified rETX (0 ~ 33 μM) was added to the 5% red blood cell solution (final concentration of 3.3%) and incubated up to 1 h at 37°C with constant swirling (300 rpm), or the purified rETX (0.2 μM) was added at a concentration leading to 50% hemolysis after 60 min with or without antagonists. Then the 1.5 ml tubes were centrifuged at 1,000 g for 10 min at 4°C, and the optical density at 540 nm of the supernatants was determined as a measure of the released hemoglobin. Relative hemolysis was shown in each figure. Relative hemolysis means the relative value compared to a control, which is defined as 1. The control of each figures varied. In general, the value of the maximal hemolysis of each figure is defined as 1.

### Scanning electron microscopy

For scanning electron microscopy observations, 5% erythrocyte solution was incubated with 300 μl of toxins (0.2 μM) at 37°C over a time course from 0 to 40 min. The samples were immediately fixed in 0.1 M phosphate buffer (pH 7.4) containing 2.5% glutaraldehyde for 4 h. Then the samples were rinsed, dehydrated in graded alcohol and acetone, and baked, plated with gold. Finally, the cells and untreated control were analyzed and photographed by S-3400N scanning electron microscopy (HITACHI, Tokyo, Japan).

### Measurement of cellular ATP

The cellular ATP content was determined using an ATP assay kit (Calbiochem-Merck, Darmstadt, Germany) via luminescence according to the manufacturer’s protocol. The erythrocytes were incubated with rETX and different antagonists for 5 min or 30 min at 37°C and 400 rpm. After incubation, the compound was centrifuged at 1,000 g for 10 min at 4°C, and the supernatant was aspirated. To estimate the release of ATP from erythrocytes, the supernatant of the RBCs (50 μl) was mixed with 50 μl of nucleotide releasing buffer and 1 μl of ATP monitoring enzyme. The sample was read using a Varioskan Flash Multimode Reader (Thermo Fisher Scientific, Waltham, MA, United States) and the software SkanIt in luminescence mode.

### Immunoblotting and oligomerisation assay

Human erythrocytes were isolated and washed with 0.01 M PBS, then lysed four times with 0.01 M PB. After each wash, the suspension was centrifuged at 15,000 g for 15 min at 4°C and the supernatant was gently removed. The pellet was incubated with rETX at 37°C for 1 h and then spun as above. The pellet was dissolved in 1% TritonX-100, separated by electrophoresis using 12% SDS-PAGE, and transferred to a PVDF membrane (Solarbio, Beijing, China). Nonspecific protein binding was reduced by incubation with 3% skim milk powder in 0.01 M PBS at room temperature for 3 h. The PVDF membrane was washed four times using PBS with 0.05% Tween-20 and incubated with primary antibody (anti-ETX/His monoclonal antibody) at 4°C overnight. The PVDF membrane was washed three times and incubated for 1 h at room temperature with a HRP-coupled goat anti-mouse IgG antibody.

### Binding of rETX to the surface of RBCs

1.25% erythrocyte solution was incubated for 15 min at 37°C with 1.0 μM mScarlet or mScarlet-rETX. Then, the RBCs were washed three times with 0.01 M PBS, the washed pellets were dissolved in 100 μl 0.01 M PBS. The fluorescence signals of erythrocytes were determined by Varioskan Flash (Thermo Fisher Scientific Waltham, MA, United States) with excitation at 569 nm and emission was detected at 600 nm.

### MTS assay

MDCK cells (3–4 × 10^4^ cells/well) were grown in 96-well plates with Dulbecco’s modified Eagle medium (DMEM) supplemented with 10% fetal bovine serum (FBS) and cultured in a 5% CO2 incubator for 24 h at 37_C. The toxin protein used in this assay was freshly purified. MTS (3-(4, 5-dimethylthiazol-2-yl) -5(3-carboxymethoxyphenyl) -2-(4-sulfopheny)-2H-tetrazolium, inner salt) (Promega, United States) assay was performed as described previously [20].
