# Supplementary material for: Hemolysis in human erythrocytes by Clostridium perfringens epsilon toxin requires activation of P2 receptors
Source: Virulence. 2018 Oct 2;9(1):1601–14. doi: 10.1080/21505594.2018.1528842 (PMC6276848; doi:10.1080/21505594.2018.1528842)
Supplement: Supplemental Material [file kvir-09-01-1528842-s001.docx]

**Supporting Information**


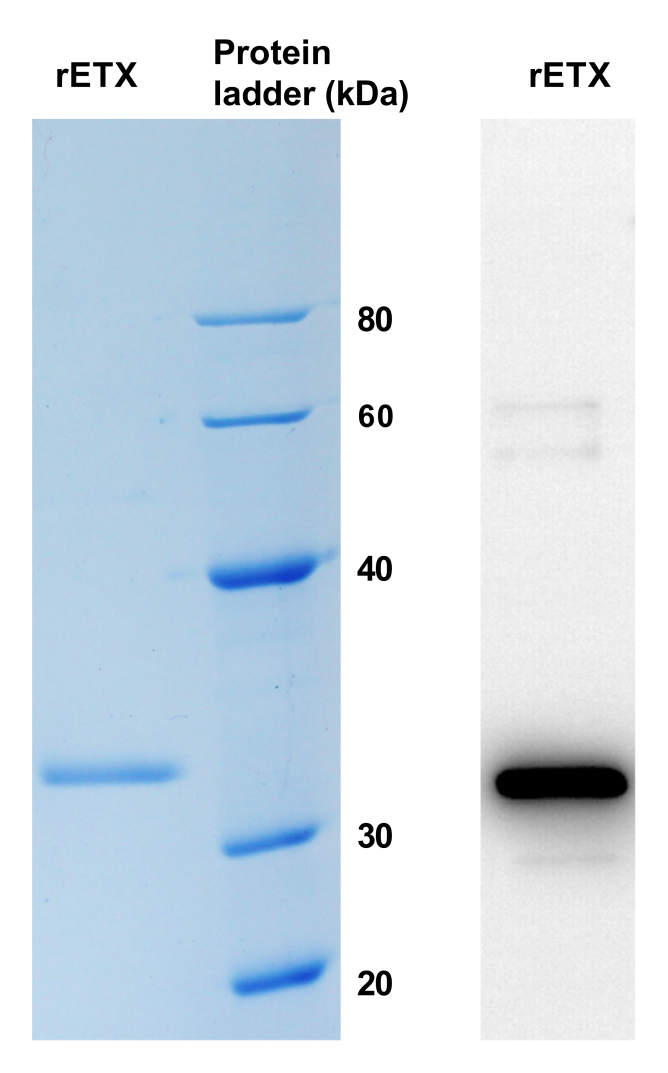


**S1 Fig. Electrophoretic gel images and immunoblotting of rETX.** Purified proteins, diluted in PBS, were heat denatured at 100°C for 5 min and electrophoresed on a 12% SDS polyacrylamide gel, and stained by Coomassie brilliant blue (left). The proteins were further analyzed by immunoblotting using an anti-ETX monoclonal antibody followed by a HRP-coupled goat anti-mouse IgG antibody (right).


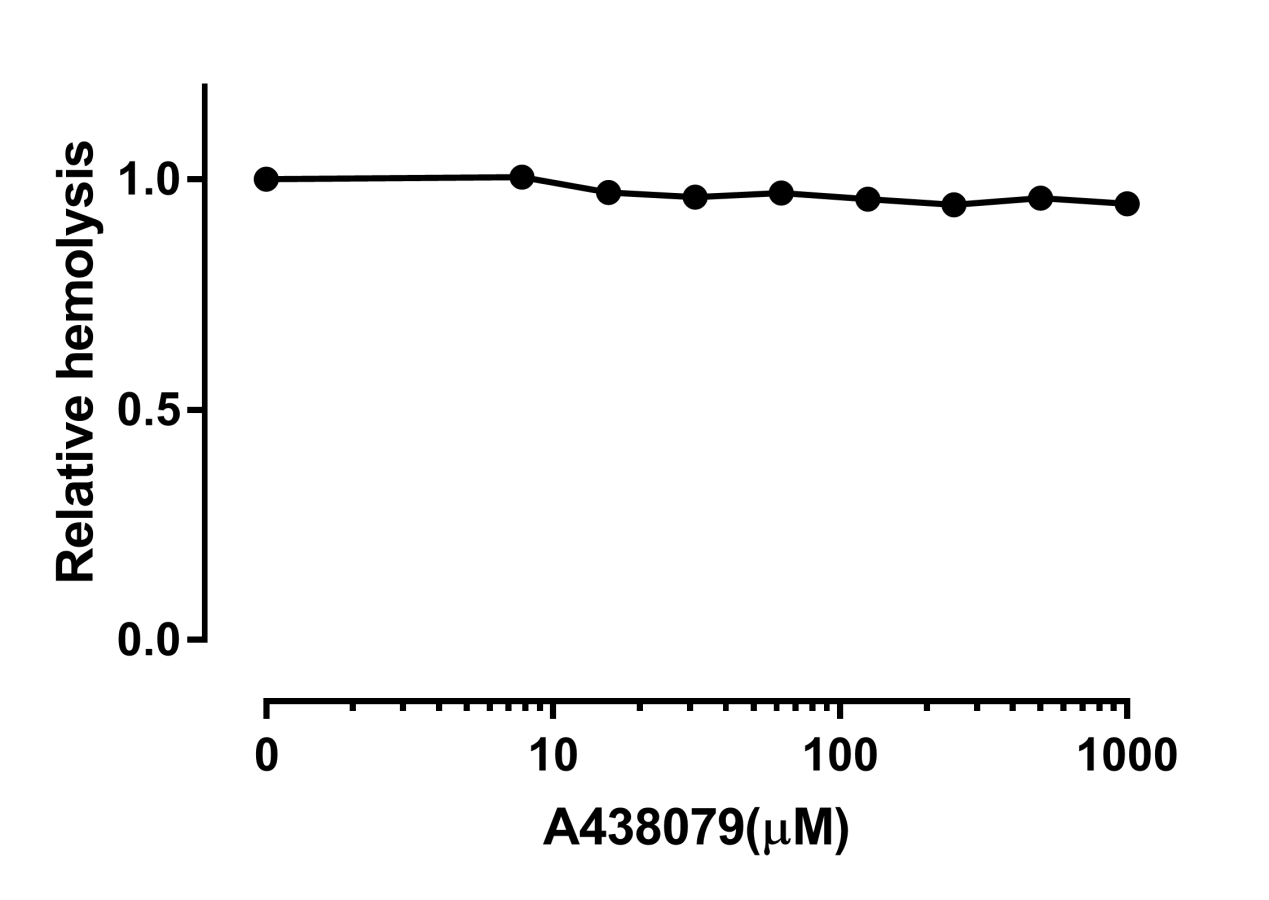


**S2 Fig. Effect of the P2X7 receptor antagonist A438079 on ETX-induced hemolysis.** Values are the mean ± SD (n=3). Hemolysis value corresponding to 0 μM of each antagonist is defined as 1.


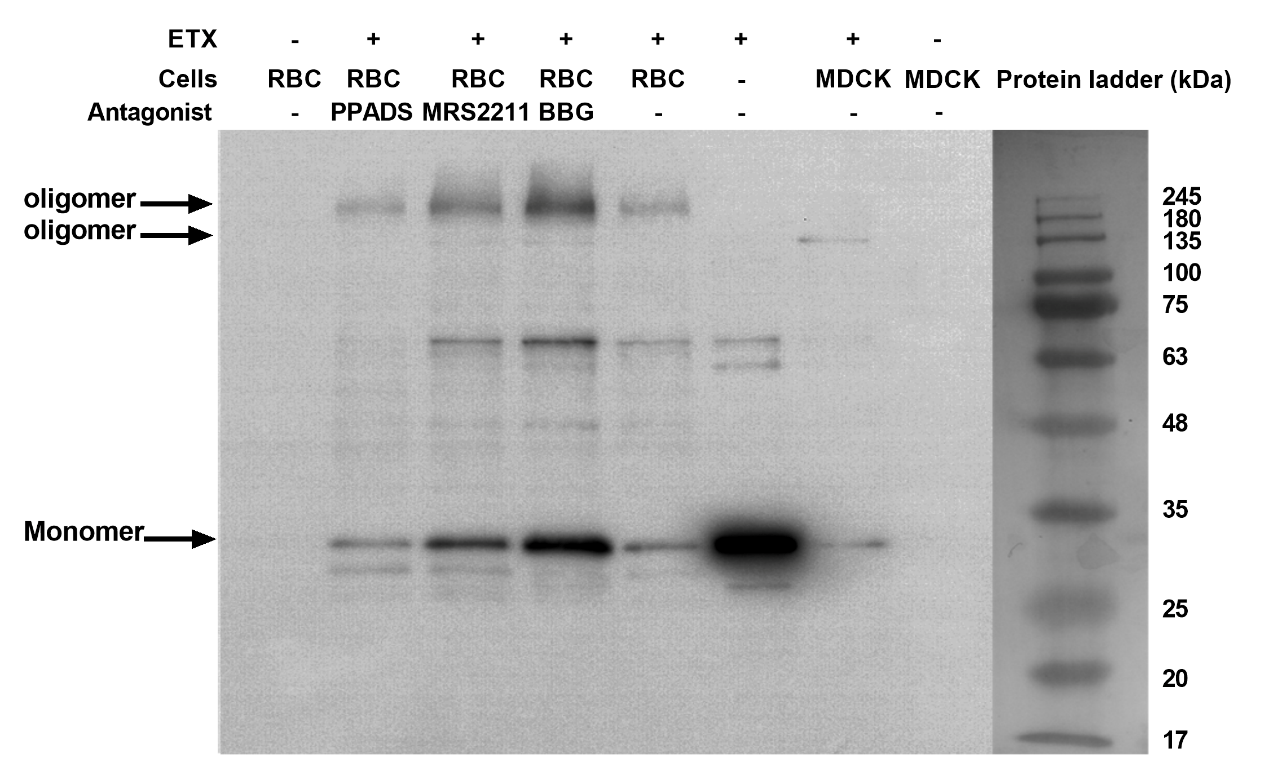


**S3 Fig. Analysis of complex formation by rETX in the membrane of human RBCs and MDCK cells.** Human RBCs and MDCK cells were treated with 200 μl of toxins (1.0 μM) for 1 h at 37°C with or without PPADS (1 mM), MRS2211 (500 μM), and BBG (500 μM). Samples were solubilized, and analyzed by SDS-PAGE, and then immunoblotted with an anti-His monoclonal antibody and a HRP-coupled goat anti-mouse IgG antibody (1: 50,000). The result was photographed using an AE-1000 cool CCD image analyzer.


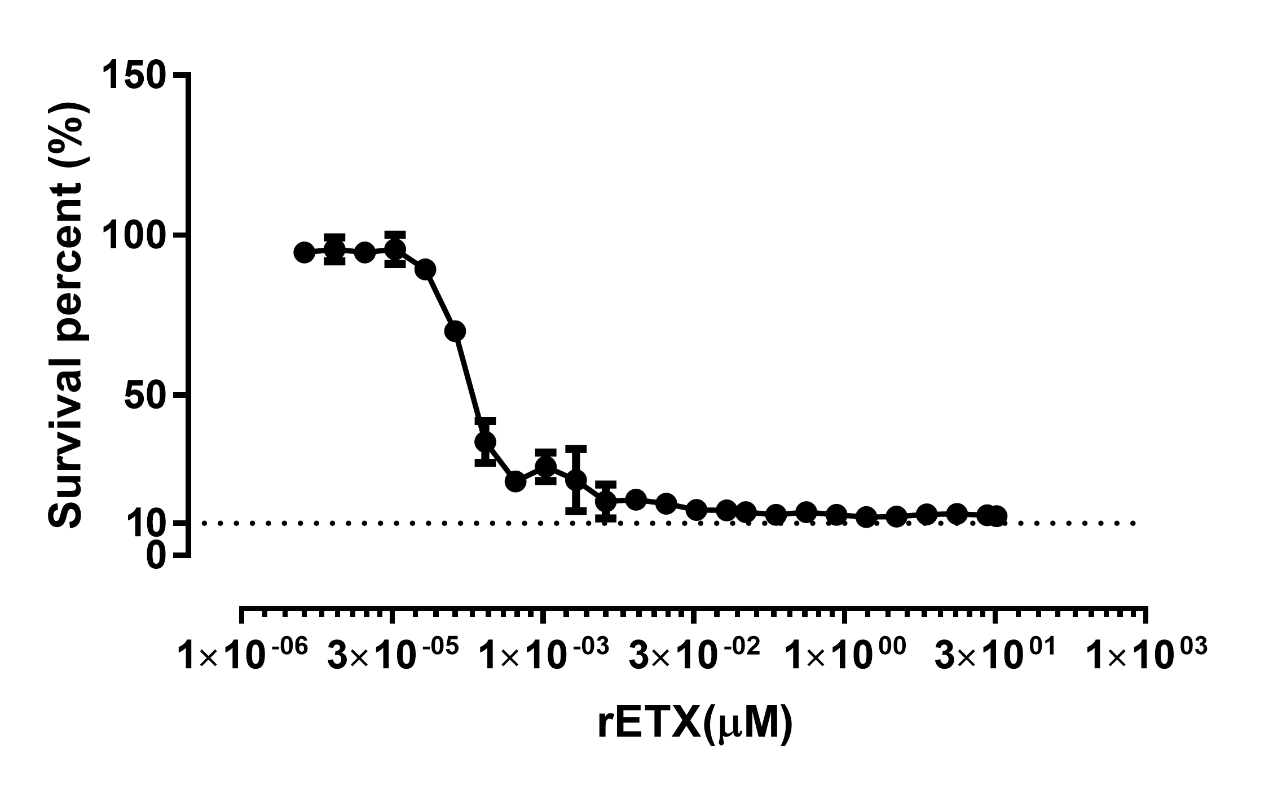


**S4 Fig.** **Cytotoxicity of rETX toward MDCK cells.** 100 μL of serially diluted toxins were added to a 96-well plate and PBS was used as a control, the viability of MDCK was detected using MTS assay.


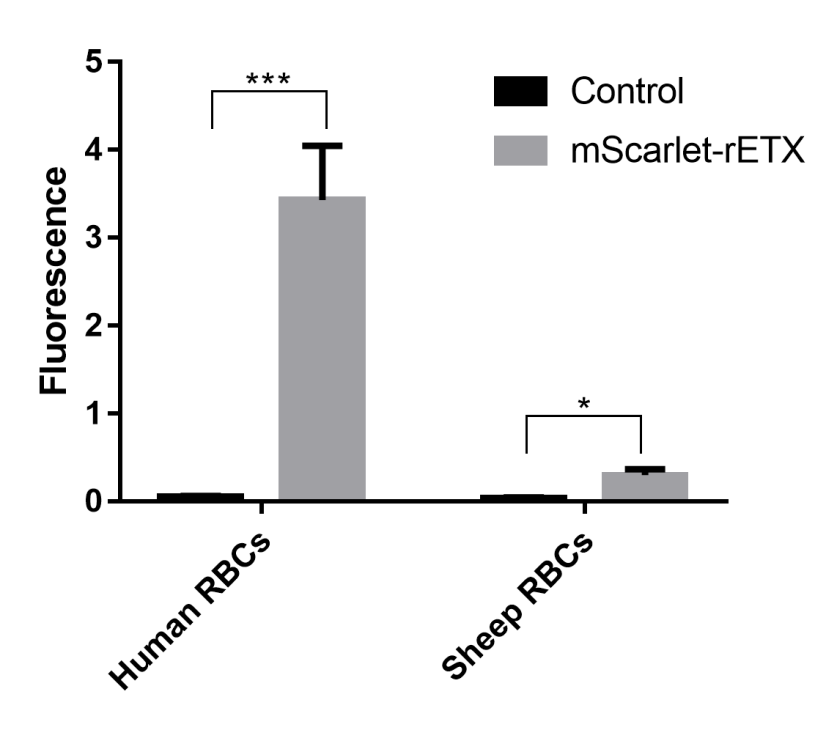


**S5 Fig. Binding of rETX to the surface of human RBCs and sheep RBCs.** A 1.25% solution of human RBCs or sheep RBCs solution was treated with 1.0 μM mScarlet or mScarlet-rETX at 37°C. Then, the RBCs were washed three times with PBS and the pellets were resuspended with 100 μl PBS. The fluorescence signals were read using Varioskan Flash (Thermo Fisher Scientific Waltham, MA, United States) with excitation at 569 nm and emission was detected at 600 nm. Values are the mean ± SD (n=3). Data were analyzed using the GraphPad Prism (Version 7) software and statistical significance was assessed by a two-tailed Student’s t-test. *p<0.05; ***p<0.001.

**
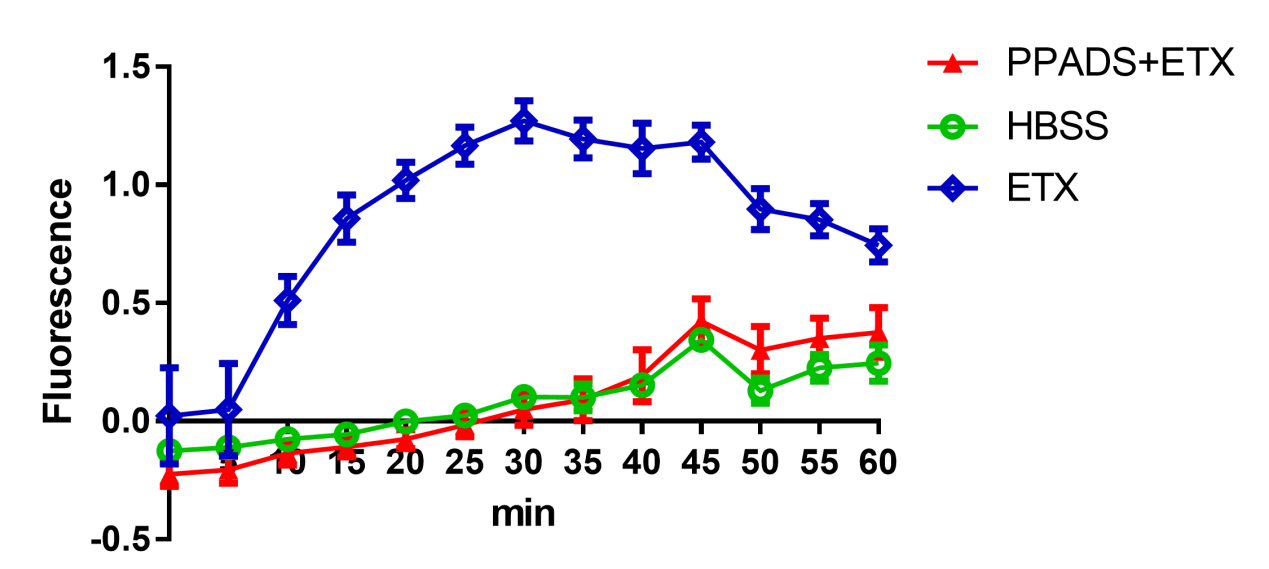
**

**S6 Fig. PPADS inhibits the ETX-induced increase in intracellular Ca^2+^.** RBCs in 1.5-ml tubes were incubated with 0.1 ml HBSS (5 mM KCl, 6 mM glucose, 12 mM MgCl_2_, 125 mM NaCl, 25 mM HEPES, pH 7.5) containing 4 mM Fluo-8L AM (AAT Bioquest, United States) in a 5% CO_2_ incubator for 1 h at 37°C. The RBCs were washed 3 times with 0.2 mL of HBSS, and then 0.2 μM of rETX diluted with HBSS, with or without PPADS, were added to the tubes. Fluorescence signals associated with Ca^2+^ concentrations were measured at 5 min intervals over 60 min using a Varioskan Flash Multiplate Reader (Thermo Fisher Scientific, Waltham, MA, United States). Values are the mean ± SD (n=3).


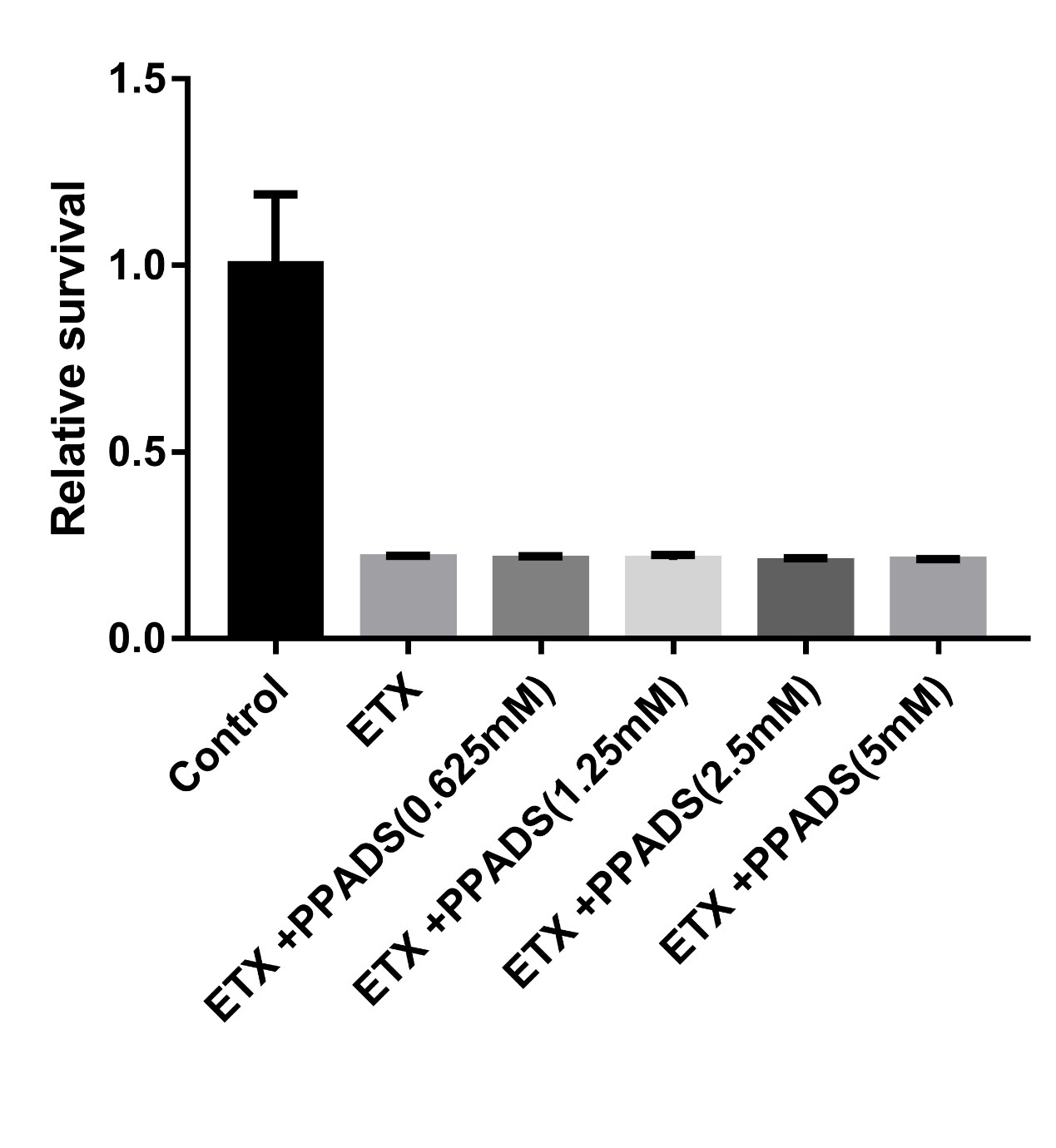


**S7 Fig. Effect of PPADS on ETX-induced death in MDCK cells.** MDCK cells (3–4×10^4^ cells/mL) were seeded in a 96-well plate with DMEM containing 10% FBS for 24 h at 37°C. Monolayers were mixed with 0.2 μM of rETX with or without serially diluted PPADS (0.625 mM–5 mM) and incubated at 37°C for 24 h. The control group was mixed with PBS. Cytotoxicity was measured by an MTS assay. Values are the mean ± SD (n=3).
